# Antimicrobial and antiparasitic potential of lupeol: antifungal effect on the Candida parapsilosis species complex and nematicidal activity against Caenorhabditis elegans

**DOI:** 10.1099/jmm.0.001976

**Published:** 2025-03-07

**Authors:** Marrie da Silva Dutra, Paulo Ricardo Monteiro Araújo, Maria Gleiciane da Rocha, Vinícius Carvalho Pereira, Alyne Soares Freitas, Raissa Geovanna Pereira Lopes, Pedro Filho Noronha Souza, Raquel Carvalho Montenegro, Waldemiro de Aquino Pereira-Neto, Géssica dos Santos Araújo, Rossana de Aguiar Cordeiro, José Júlio Costa Sidrim, Glaucia Morgana de Melo Guedes, Débora de Souza Collares Maia Castelo-Branco, Marcos Fábio Gadelha Rocha

**Affiliations:** 1 School of Veterinary Medicine, Postgraduate Program in Veterinary Sciences, State University of Ceará, Fortaleza, Ceará, Brazil; 2 Department of Pathology and Legal Medicine, Postgraduate Program in Medical Microbiology, Specialized Medical Mycology Center, Federal University of Ceará, Fortaleza, Ceará, Brazil; 3 Drug Research and Development Center, Federal University of Ceará, Fortaleza, Ceará, Brazil; 4 Department of Transport Engineering, Federal University of Ceará, Fortaleza, Ceará, Brazil

**Keywords:** triterpenoid, yeast, nematode, inhibition, *in vitro*, sterol 14α-demethylase

## Abstract

**Introduction.** There is growing concern about infections caused by non-*albicans Candida* species, including species of the *Candida parapsilosis* complex – which have seen a considerable increase in cases during the COVID-19 pandemic – in addition to concern about nematode resistance to currently used anthelmintics.

**Gap Statement.** Lupeol is a triterpenoid phytosterol that has a wide range of biological activities, although its antifungal and antiparasitic potential is still poorly explored. Additionally, its effect on the biofilm of the *C. parapsilosis* species complex has not yet been studied.

**Aim.** This study aimed to investigate the antifungal effect of lupeol against *C. parapsilosis* complex species, in planktonic cells and mature biofilms, as well as its nematicidal potential against *Caenorhabditis elegans*. In addition, molecular docking was performed to identify potential target molecules for lupeol’s antifungal effect.

**Methodology.** Twelve strains of *C. parapsilosis* species complex were used. Planktonic susceptibility was performed through the broth microdilution assay, while the antibiofilm effect was investigated by measuring the biomass and metabolic activity. The antifungal mechanism of action of lupeol was investigated by target fishing. The evaluation of the nematicidal effect was performed using the *C. elegans* infection model.

**Results.** Lupeol demonstrated antifungal activity against planktonic cells with a MIC between 64 and 512 µg ml^−1^. In mature biofilms, lupeol was able to reduce biomass starting from a concentration of 1024 µg ml^−1^ and reduce metabolic activity from a concentration of 64 µg ml^−1^. It was observed that there was interaction of lupeol with the enzyme 14α-demethylase. Furthermore, lupeol had a nematicidal effect from a concentration of 64 µg ml^−1^.

**Conclusion.** Lupeol exhibits an antifungal effect on the *C. parapsilosis* species complex, in the planktonic and mature biofilm forms, possibly by affecting the ergosterol synthesis. Lupeol further demonstrated a nematicidal potential.

## Introduction

Worldwide, candidemia or invasive candidiasis affects ~1.5 million people, causing 995 000 deaths (63.6%) [1]. Invasive candidiasis is one of the most serious community and nosocomial infections, and although *Candida albicans* is still the most frequently isolated species, a significant increase in infections caused by non-*albicans* species has been observed in recent years [2,4]. During the COVID-19 pandemic, the incidence of candidemia increased substantially, two to six times compared with the pre-pandemic period, with predominance of infections caused by non-*albicans Candida* species, while species of the *Candida parapsilosis* complex were among the most commonly isolated [5,6]. Different studies have shown an increase in the incidence of *C. parapsilosis* infections [7,8]. In addition, in the same period, an increase in the frequency of *C. parapsilosis* isolates resistant to fluconazole has also been observed [9,11].

The *C. parapsilosis* complex includes three cryptic species: *C. parapsilosis sensu stricto, C. metapsilosis* and *C. orthopsilosis*, differentiated only through molecular diagnostics [12]. These three species can cause a range of clinical presentations, from colonization to superficial and disseminated infections, especially with the potential to cause outbreaks in hospitals [13,14]. The therapeutic options available for the treatment of invasive candidiasis are limited. The three main classes of antifungals used are polyenes (amphotericin B), azole derivatives (fluconazole, itraconazole and voriconazole) and echinocandins (caspofungin) [15,16]. However, infections by *Candida spp*. often present therapeutic failure, mainly as a result of antifungal resistance, caused by various mechanisms, including biofilm formation, which confers greater resistance to conventional antifungal therapies since biofilm-producing strains show a significant increase in resistance to antifungal drugs and the host immunity [17]. Therefore, the search for new compounds that have antifungal activity against these yeasts is essential.

Lupeol (3β-hydroxy-20(29)-lupene) is a biologically active triterpenoid found in a variety of plant species, such as *Aloe vera*, *Aloe barbandesis* Miller, *Brassica oleracea var*. *capitata* and *Olea europaea*, which have therapeutic potential [18,19]. In recent years, studies have shown that this compound has anti-inflammatory, anticancer, antimicrobial, antiprotozoal and antiviral activities [18,21]. However, research evaluating its antifungal activity against the *C. parapsilosis* species complex and nematodes is still scarce.

Nematodes are a group of parasites that cause a major health problem to livestock worldwide, leading to significant economic losses to producers, especially those of ruminants [22]. The main classes of anthelmintics are macrolide lactones, benzimidazoles and nicotinic agonists [23]. In recent decades, the intensive use of anthelmintics has led to a high level of resistance in these parasites [23,25]. In Brazil, different studies have indicated the occurrence of resistance to all classes of anthelmintics used [25,26]. In addition, multiple resistance of the main genera of parasites – *Haemonchus*, *Trichostrongylus* and *Oesophagostomum* – against the usual anthelmintics has also been observed [25]. Given this challenge of parasite resistance, the need to search for new compounds with nematicidal activity that allow their applicability in the control of nematodes is necessary.

Thus, this study aimed to investigate the antifungal effect of lupeol against *C. parapsilosis* complex species, in planktonic cells and mature biofilms, as well as its nematicidal potential against *Caenorhabditis elegans*. Additionally, molecular docking was performed to identify potential target molecules for lupeol’s antifungal effect.

## Methods

### Micro-organisms

Eleven strains of the *C. parapsilosis* complex species (four *C*. *parapsilosis sensu stricto*, four *C*. *orthopsilosis* and three *C*. *metapsilosis*) were used, eight of veterinary origin and three isolated from humans. The collection site/host from where the species of the *C. parapsilosis* complex were isolated is presented in Table 1. In addition, the strain *C. parapsilosis* ATCC 22019 was included. The strains belong to the fungal collection of the Specialized Medical Mycology Center of the Federal University of Ceará. The identification of the species was previously confirmed through the analysis of their macro and micromorphological characteristics and molecular methods (PCR based on restriction analysis of PCR products of the Secondary Alcohol Dehydrogenase (SADH) gene, using the restriction enzyme BanI) (New England Biolabs, USA) [12,27, 28]. The micro-organisms were cultured in potato dextrose agar (HiMedia, India) at 25 °C for 48 h before performing the experiments.

**Table 1.** Collection site/host and antifungal susceptibility of planktonic cells of the *C. parapsilosis* species complex

Strains	Collection site/host	*C. parapsilosis* complex	Lupeol	Amphotericin B	Fluconazole	Itraconazole	Voriconazole	Caspofungin
			MIC (µg ml^–1^)					
01-1-214	Mouth/bat	*C. parapsilosissensu stricto*	256	0.062	0.25	0.0155	<0.03125	0.125
CEMM3H	nr	*C. parapsilosissensu stricto*	256	0.0625	0.5	0.03125	0.03125	1
01-1-196	Eye/equine	*C. parapsilosissensu stricto*	256	0.062	1	0.0155	0.031	0.125
01-1-186	Cloaca/parrot	*C. parapsilosissensu stricto*	256	0.25	0.5	0.031	<0.03	0.5
05-5-092	Blood culture/human	*C. orthopsilosis*	256	0.062	4	0.25	0.062	1
01-1-200	Nose/equine	*C. orthopsilosis*	256	0.062	1	0.062	0.125	0.25
01-1-178	Faeces/parrot	*C. orthopsilosis*	64	0.062	2	0.125	0.125	0.25
01-1-210	Anus/bat	*C. orthopsilosis*	256	0.062	1	0.062	0.062	0.25
01-1-175	nr	*C. metapsilosis*	128	0.062	2	0.062	0.031	0.125
01-1-167	Blood culture/human	*C. metapsilosis*	256	0.125	2	0.062	0.031	0.25
01-1-199	Nose/equine	*C. metapsilosis*	256	0.0155	2	0.0155	<0.03125	0.25
01-1-217	Faeces/human	C. parapsilosissensu stricto*	512	0.25	2	0.062	–	0.5

nd, Not reported.

* *C. parapsilosis* ATCC 22019.

### Lupeol and antifungals

The lupeol stock solution (Sigma-Aldrich, St. Louis, USA) was obtained by diluting the compound in DMSO (100%) to obtain a final concentration of 23 500 µg ml^−1^. The final DMSO concentration used in the tests did not influence fungal growth. Amphotericin B (AMB, Sigma Chemical Corporation, USA) was selected as the standard antifungal to be used in the control of planktonic susceptibility. It was diluted in DMSO to produce the stock solution with a final concentration of 1600 µg ml^−1^. In addition, antifungal drugs were used to determine the susceptibility profile of the strains evaluated, with stock solutions having concentrations of 6400 µg ml^−1^ for fluconazole (FLU, Sigma Chemical Corporation, USA), 1600 µg ml^−1^ for itraconazole (ITR, Sigma Chemical Corporation, USA), voriconazole (VOR, Sigma Chemical Corporation, USA) and caspofungin (CAS, Sigma Chemical Corporation, USA). Lupeol (PubChem CID: 259846) was kept at 2–8 °C and the antifungal drugs at −20 °C until use.

### Antifungal susceptibility of planktonic *C. parapsilosis* species complex

Antifungal susceptibility testing of the *C. parapsilosis* species complex was performed using the broth microdilution technique, as established by document M27 issued by the Clinical and Laboratory Standards Institute [29]. Lupeol (0.5–512 µg ml^−1^), amphotericin B (0.0313–16 µg ml^−1^), fluconazole (0.125–64 µg ml^−1^), itraconazole (0.0313–16 µg ml^−1^), voriconazole (0.0313–16 µg ml^−1^) and caspofungin (0.015–8 µg ml^−1^) were used. The MIC values were defined as the lowest concentration capable of inhibiting fungal growth by 50%, in comparison with the control without drug, for lupeol, 100% for amphotericin B and 50% for fluconazole, itraconazole, voriconazole and caspofungin.

Microdilution assays were performed in 96-well plates with a final volume of 200 µg ml^−1^. Inocula were prepared at a turbidity of 0.5 on the McFarland scale in saline and dilute in Roswell Park Memorial Institute (RPMI 1640) medium to a final concentration from 5.0×10^2^ to 2.5×10^3^ cells per ml. The plates were incubated at 35 °C, and visual readings of fungal growth inhibition were performed after 24 and 48 h. All assays were performed in duplicate for each strain at three different times, in addition to the inclusion of growth control and sterility control [29].

### Biofilm formation and antibiofilm effect of lupeol

Six strains were selected to evaluate the antibiofilm effect of lupeol (three *C*. *parapsilosis sensu stricto*, one *C*. *metapsilosis* and two *C*. *orthopsilosis*). After establishing the lupeol MIC, its activity on mature biofilms of the *C. parapsilosis* species complex was evaluated at concentrations of 4xMIC (1024 µg ml^−1^), 2xMIC (512 µg ml^−1^), MIC (256 µg ml^−1^), MIC/2 (128 µg ml^–1^) and MIC/4 (64 µg ml^–1^). Biofilm formation and susceptibility evaluation were performed as described by Brilhante *et al*. [30]. For biofilm formation, the yeasts were previously cultured in potato dextrose agar (HiMedia, India) at 35 °C for 48 h and then resuspended in RPMI 1640 medium until reaching turbidity of 1 on the McFarland scale (1×10^6^ cells per ml). Subsequently, 200 µl of each inoculum was transferred to a 96-well flat-bottomed polystyrene plate and incubated at 37 °C for 48 h. After biofilm maturation, the medium was removed and each well was washed twice with sterile PBS to remove non-adhered cells. Subsequently, 200 µl aliquots of RPMI 1640 medium containing serial dilutions of lupeol (1024–64 µg ml^−1^) were added to the wells containing the mature biofilms and immediately incubated at 35 °C for 48 h.

To quantify the biomass production of mature biofilms, 48 h after the addition of lupeol, the staining assay with 0.3% crystal violet was performed as described by Brilhante *et al*. [30]. The wells containing biofilms were washed twice with sterile PBS, fixed in 100% methanol and stained with 0.3% crystal violet. Then the biofilms were washed with distilled water and destained with a 33% acetic acid solution. The volumes were transferred to the wells of a new microplate, and the OD of acetic acid was immediately read with a spectrophotometer at 540 nm. The metabolic activity of mature biofilms was quantified 48 h after exposure to lupeol through the yellow tetrazolium salt reduction assay [3-(4,5-dimethyl-thiazol-2-yl)-2,5-diphenyltetrazolium bromide] (MTT), as described by Brilhante *et al*. [31]. For this purpose, 100 µl aliquots of the MTT solution (0.5 mg ml^−1^) prepared in PBS, together with 2% glucose and filtered through a 0.22 µM membrane, were added to the wells, and the plates were incubated while protected from light for 3 h at 35 °C. After incubation, the MTT solution was removed and 100 µl of DMSO was added to each well. The plate was again incubated for 30 min under agitation, protected from light. Finally, the DMSO solution was transferred to the wells of a new flat-bottom plate, and the reading was performed with a spectrophotometer at 690 nm.

The effect against mature biofilm cells was identified when a reduction in biomass and metabolic activity of biofilms treated with lupeol was observed in comparison with the biofilm growth control. All experiments were performed in duplicate for each strain at three different times, and the *C. parapsilosis* ATCC 22019 strain was used as a positive control. For all strains, growth controls without the addition of lupeol were used, in addition to sterility control [31].

Confocal laser scanning microscopy (CLSM) (Nikon, Tokyo, Japan) was used for the structural analysis of mature biofilms [32], both unexposed and exposed to lupeol. Two isolates (*C. parapsilosis sensu stricto* and *C. orthopsilosis*), best biofilm formers, were selected for the CLSM methodology and subsequent analysis. The mature biofilms were grown directly on thermanox coverslips, using 24-well tissue culture plates. After 48 h of growth, the coverslips were washed twice with PBS, and then lupeol at 4xMIC (1024 µg ml^−1^) was added to the samples. Unexposed growth controls were incubated in RPMI 1640 only. Plates were then incubated for a further 48 h at 35 °C, after which the mature biofilms were washed twice with PBS and stained with the LIVE/DEAD^®^ FungaLight^™^ Yeast Viability Kit (Invitrogen, USA) at a dilution of 1.5 µl to 1.0 ml of sterile distilled water. Coverslips with biofilms were evaluated using a Nikon C2 confocal microscope at 488 nm to identify viable yeasts (green staining) and 561 nm for damaged cells (red staining). For image analysis, five equidistant points were selected and three-dimensional images were taken for colourimetric quantification and Z-slice measurement using the Image J 1.50 software (National Institutes of Health, Wisconsin, USA). The structural parameters of the biomass, such as roughness coefficient, maximum thickness and average thickness, were analysed using the Comstat2 TM software (MATLAB^©^, Natick, USA) [32]. Biomass is the total amount of material adhered to the biofilm cells, while average thickness is the parameter that assesses the number of cell layers in conjunction with the biomass, evaluating the biofilm’s detachment capacity and fragmentation.

### Evaluation of the mechanism of action of lupeol against the *C. parapsilosis* species complex by molecular docking

#### Target fishing for lupeol

The SMILES lupeol sequence was obtained from PubChem (https://pubchem.ncbi.nlm.nih.gov/), a database that provides chemical information for more than 100 million compounds [33].

The search for targets was performed via the TargetNet online server (http://targetnet.scbdd.com/home/index/), but the database of this web server does not contain fungal structures in its search arsenal. For this reason, from the targets listed with high probability of binding (1.0), protein–protein blast analyses were performed at the National Center for Biotechnology Information (NCBI) (https://blast.ncbi.nlm.nih.gov/Blast.cgi) to search for *C. parapsilosis* proteins similar to the targets described in TargetNet.

#### Molecular docking

The 3D structure of 14α-demethylase was determined using AlphaFold, a program that uses artificial intelligence (AI) to predict the three-dimensional structure of proteins from their amino acid sequence [34]. The structure was validated by Verify3D through the SAVES v6.1 server (https://saves.mbi.ucla.edu/). The 3D structure of lupeol was obtained from the PubChem database (https://pubchem.ncbi.nlm.nih.gov/).

Molecular docking assays were performed with DockThor (https://dockthor.lncc.br/v2/), a free online protein–ligand docking server launched in 2013 [35]. The docking box position followed the following parameters: x_center: 5.156; y_center: 1.857; z_center: −1.039; x_size: 40; y_size: 40; z_size: 40. The 3D images showing the binding poses and polar interactions were obtained using the Pymol software. The 2D representation of the polar and nonpolar interactions was obtained using the Discovery Studio software (BIOVIA).

#### Assay of the nematicidal activity

To evaluate the nematicidal activity of lupeol, nematodes of the species *C. elegans* were used as an experimental infection model, according to the protocol suggested by Brilhante *et al*. [36]. The nematodes were previously cultured in a nematode growth medium containing *Escherichia coli* OP50. The cultured nematodes underwent successive washings with M9 buffer and brain heart infusion agar broth (BHI) (Himedia, USA). Then, 50 worms at the L4 larval stage were collected and transferred to new plates containing 2 ml of a liquid medium consisting of 79% M9 buffer, 20% BHI broth, 10 mg ml^−1^ of cholesterol diluted in ethanol and 100 mg ml^−1^ of ciprofloxacin, plus different concentrations of lupeol (16–256 µg ml^−1^). The plates were incubated at 25 °C, and the viability of the nematodes was analysed at 24, 48, 72 and 96 h. The dead worms were removed from the plates throughout the 96 h of the experiment, and a survival curve was plotted at the end of the test. As a positive control, the anthelmintic levamisole (8 mg ml^−1^) was used, while *E. coli* OP50 was used as a negative control [37].

#### Statistical analysis

The results of planktonic susceptibility are presented through the MIC variable of each drug on yeasts. For the analysis of biomass and cell viability in biofilms, the absolute values of OD obtained through the crystal violet and MTT assays with a spectrophotometer were used, comparing the results of the control and the different concentrations of lupeol. Comparison between three or more groups was performed with ANOVA and Tukey’s post-test. For the analysis of nematode mortality rates, Kaplan–Meier survival curves were constructed by applying the log-rank and Breslow tests. For confocal laser scanning microscopy analyses, the Student t-test was used when the data presented symmetry, and the Mann–Whitney test was performed when the data were asymmetrical for comparison between pairs. The maximum significance level adopted for affirmative conclusions was 5%.

## Results

### Planktonic susceptibility assay demonstrated that lupeol exhibits antifungal effects against the * C. parapsilosis* species complex

The antifungal susceptibility profile of the evaluated strains is presented in Table 1. For lupeol, one strain of the species * C. orthopsilosis* exhibited a MIC of 64 µg ml^−1^ and one strain of *C. metapsilosis* presented a MIC of 128 µg ml^−1^. The other strains of the *C. parapsilosis* species complex (four *C*. *parapsilosis sensu stricto*, three *C*. *orthopsilosis* and two *C*. *metapsilosis*) presented a MIC of 256 µg ml^−1^ (*n*=9/12). The strain *C. parapsilosis* ATCC 22019 presented a MIC of 512 µg ml^−1^. In this study, no fungicidal activity (100% inhibition) of lupeol was observed at the concentrations evaluated. Regarding antifungals, the strains were sensitive to the drugs tested with the following MIC ranges: 0.0155–0.25 µg ml^−1^ for amphotericin B, 0.25–4 µg ml^−1^ for fluconazole, 0.0155–0.25 µg ml^−1^ for itraconazole, <0.03125–0.125 µg ml^−1^ for voriconazole and 0.125–1 µg ml^−1^ for caspofungin. These results indicate that lupeol showed antifungal activity against strains of the *C. parapsilosis* complex with MICs ranging from 64 to 512 µg ml^−1^.

### Lupeol exhibits antibiofilm effects against the *C. parapsilosis* species complex

**Fig. 1.**
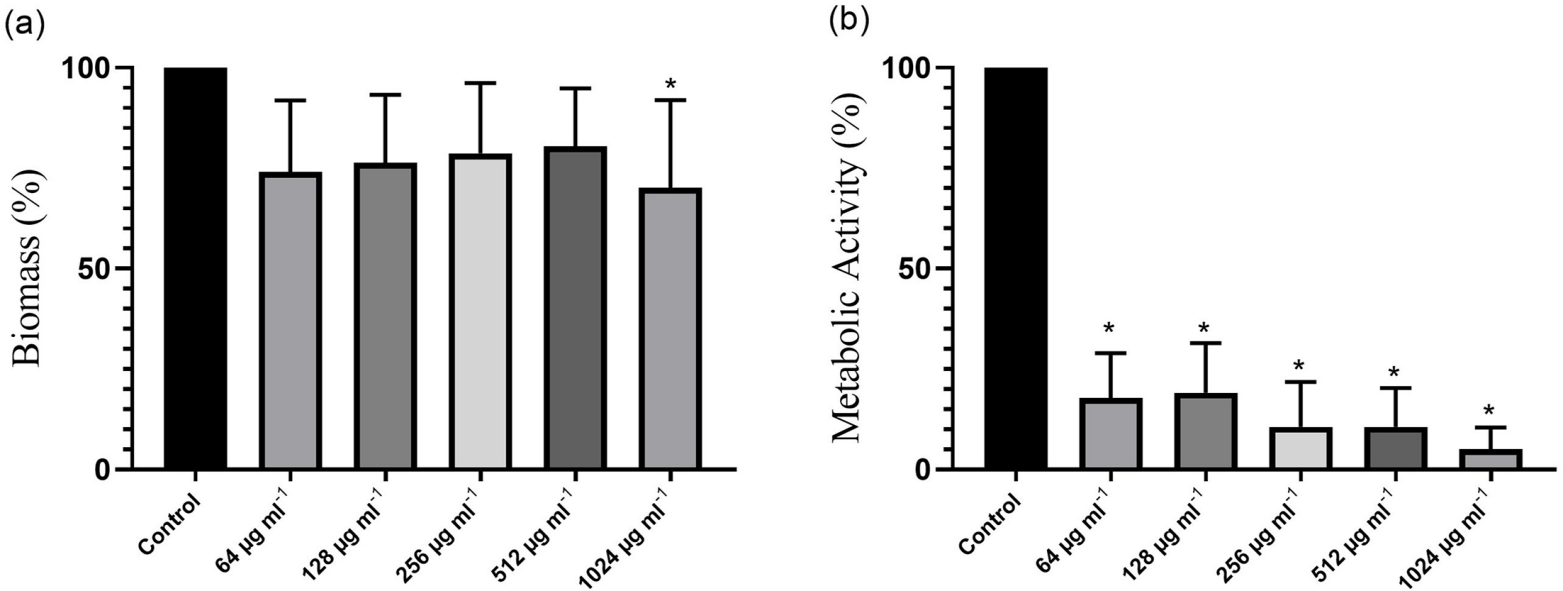
Analysis of biomass reduction by crystal violet staining (**a**) and metabolic activity by MTT reduction assay (**b**) of mature biofilms of *C. parapsilosis* complex species after exposure to the compound lupeol at different concentrations (64, 128, 256, 512 and 1024 µg ml^−1^). Biofilm biomass and metabolic activity are expressed as mean±sd of the OD obtained after exposure of mature biofilms to the *C. parapsilosis* complex at different concentrations of lupeol. *Indicates statistically significant differences between the control and the mature biofilm exposed to the compound for each concentration (*P*<0.05). *n*=6.

**Fig. 2.**
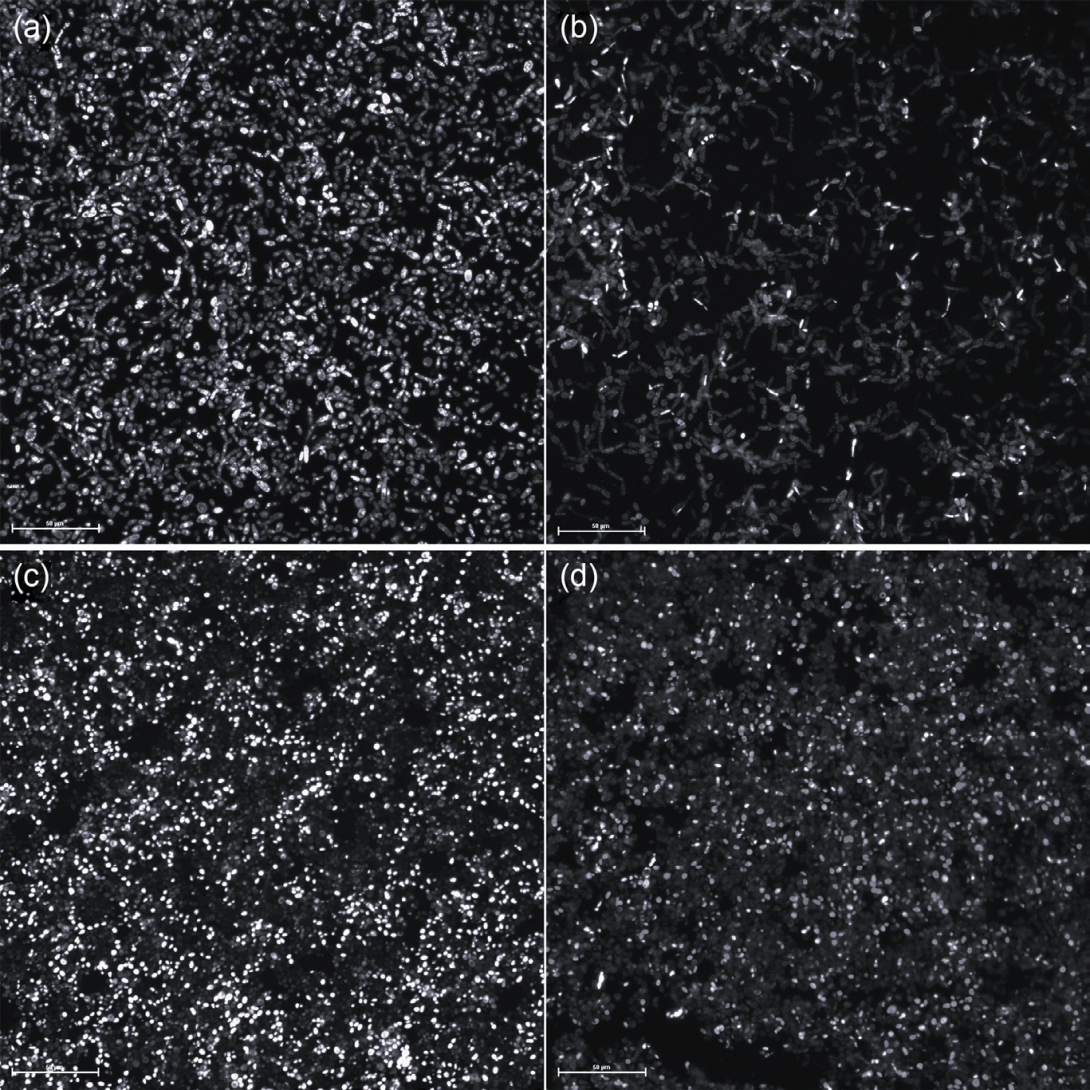
Lupeol activity against mature biofilms of *C. parapsilosis sensu stricto* and *C. orthopsilosis* analysed by confocal laser scanning microscopy on greyscale. (a) Drug-free biofilm growth of *C. parapsilosis sensu stricto*. (b) Biofilm of *C. parapsilosis sensu stricto* exposed to 1024 µg ml^−1^ of lupeol. (c) Drug-free biofilm growth of *C. orthopsilosis*. (d) Biofilm of *C. orthopsilosis* exposed to 1024 µg ml^−1^ of lupeol. These images show that the biofilms exposed to lupeol have areas with higher amounts of dead/damaged cells compared with drug-free growth control. Scale bar=50 µm. Images were acquired at 488 nm to reveal SYTO9 dye and at 561 nm to reveal propidium iodide. Magnification: 400×. *n*=2.

**Fig. 3.**
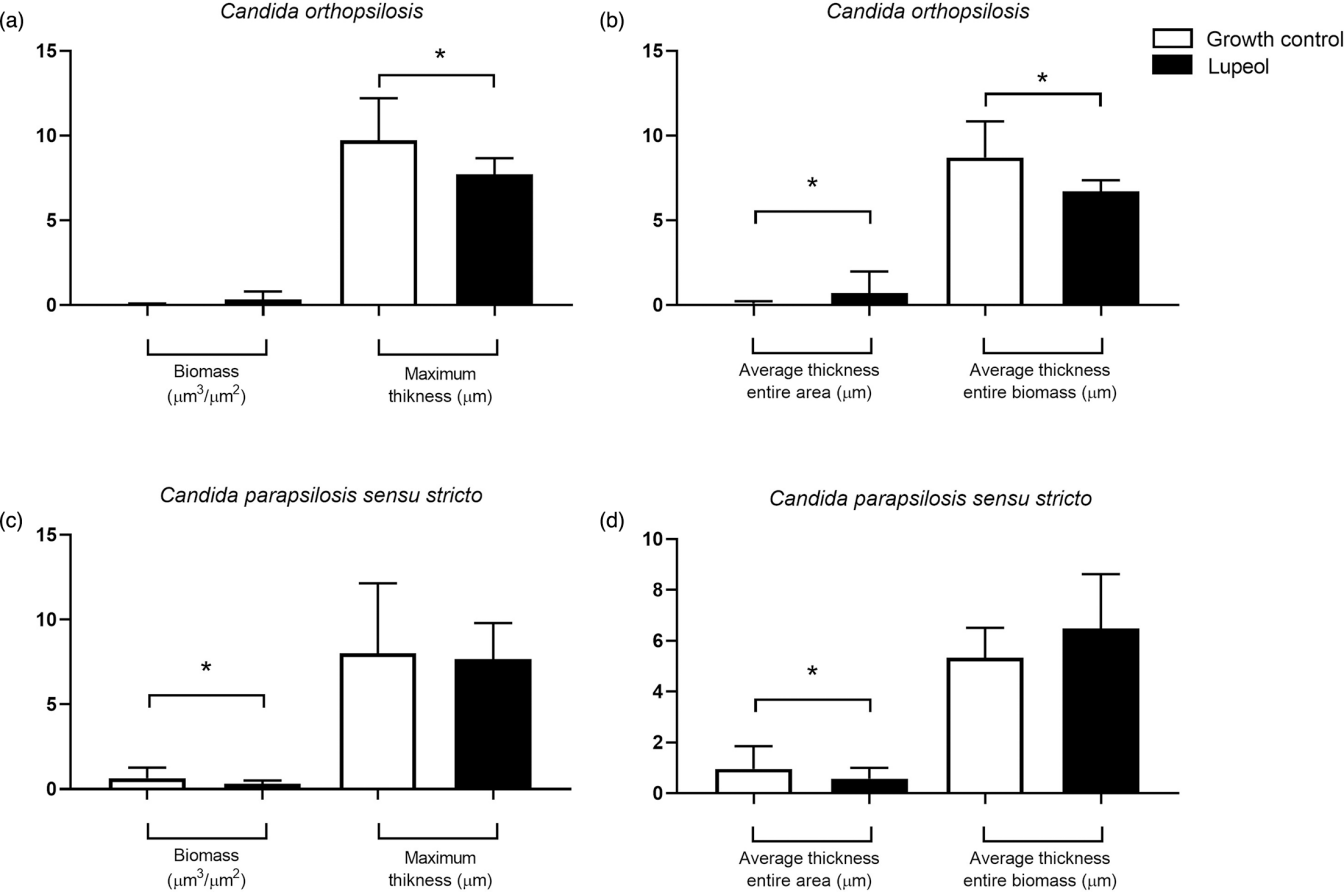
Effect of lupeol against mature biofilms of *C. parapsilosis sensu stricto* and *C. orthopsilosis*. One strain of *C. parapsilosis sensu stricto* and one strain of *C. orthopsilosis* were randomly chosen for representation. The following parameters were evaluated: biomass, maximum thickness, average thickness entire area and average thickness biomass. The data are expressed as mean±sd. (a) *C. orthopsilosis* biomass and maximum thickness. (b) *C. orthopsilosis* average thickness entire area and average thickness entire biomass. (c) *C. parapsilosis sensu stricto* biomass and maximum thickness. (d) *C. parapsilosis sensu stricto* average thickness entire area and average thickness entire biomass. *Indicates statistically significant differences between drug-free growth control and lupeol-exposed mature biofilms (*P*<0.05). *n*=2.

Exposure of the biofilms to lupeol reduced the biomass in relation to the growth control, without the addition of the compound, with a statistically significant difference at concentration 1024 µg ml^−1^ (4xMIC) (*P*=0.022). Regarding metabolic activity, lupeol had an effect at all concentrations evaluated compared to the growth control (*P*<0.0001) (Fig. 1). In the pairwise analysis, when comparing the different concentrations of lupeol used regarding the reduction in metabolic activity, a statistical difference was observed between the lowest concentrations (64 and 128 µg ml^−1^) and maximum concentration (1024 µg ml^−1^), which presented the greatest reduction in metabolic activity (*P*<0.0001). The structural analysis of the mature biofilms treated with lupeol presented areas with higher amounts of dead/damaged cells compared with control (Figs 2 and S1, available in the online Supplementary Material). After the exposure to lupeol, biofilm CLSM analyses in general revealed a decrease in biofilm biomass, but this decrease was only significant (*P*<0.05) for *C. parapsilosis sensu stricto*. For *C. orthopsilosis,* there was a decrease (*P*<0.05) in maximum thickness and medium biomass area (Fig. 3). Furthermore, exposure to lupeol significantly decreased the total average area of biofilms for both species (*P*<0.05). Thus, lupeol demonstrated antifungal potential in mature biofilms, significantly reducing metabolic activity and biomass, the latter to a lesser extent.

### Molecular docking showing that lupeol interacts with enzyme 14α-demethylase

**Fig. 4.**
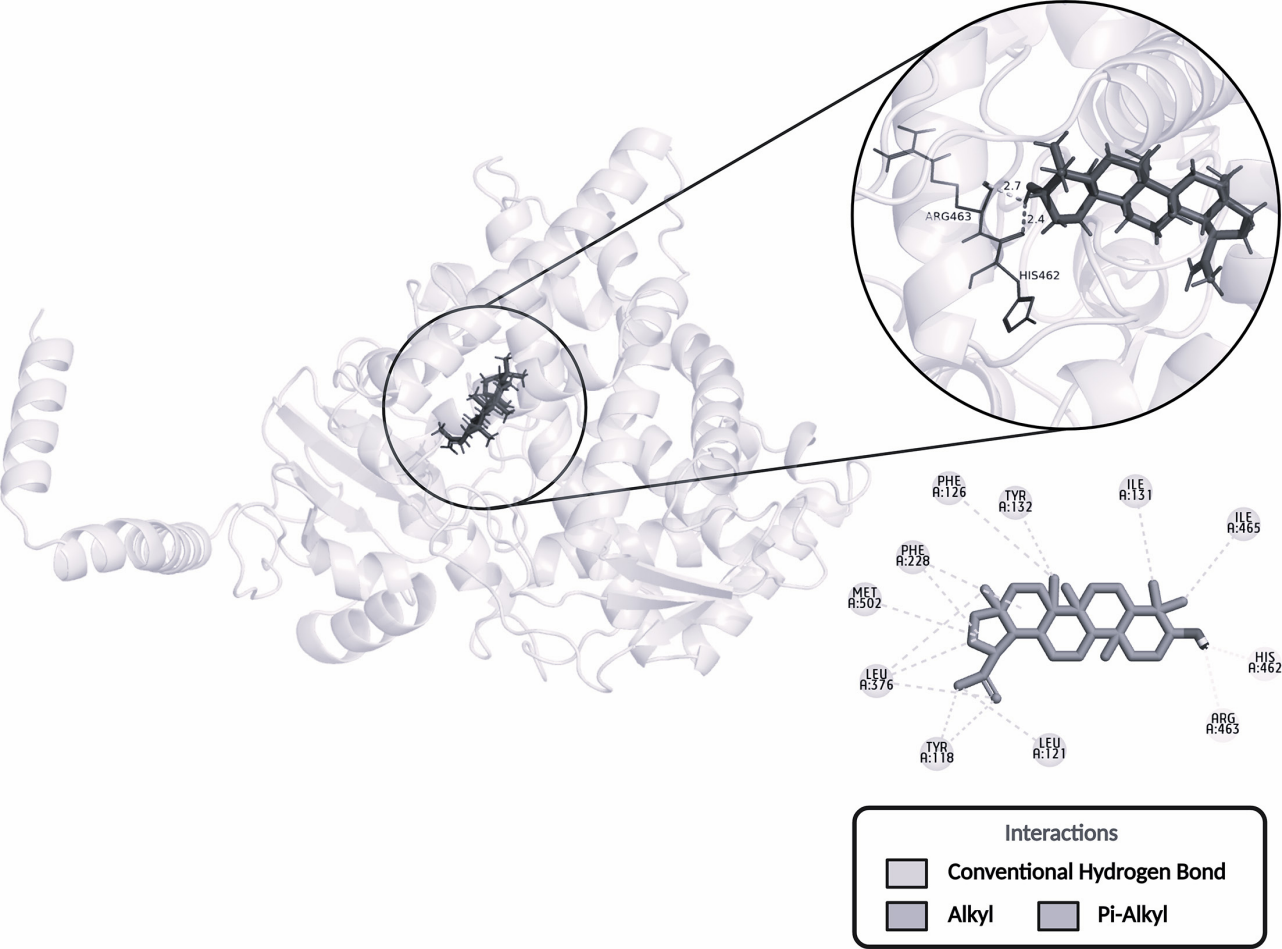
Interaction pose and polar and nonpolar bonds of lupeol with the enzyme 14α-demethylase after molecular docking assays.

Protein–protein blast analysis identified the enzyme sterol 14α-demethylase as a promising target in the fungal cell, from the human target Steroid 17-α-hydroxylase/17,20 lyase (P05093), which was selected for molecular docking analyses. The molecular docking assay presented the interaction of lupeol with the enzyme 14α-demethylase. From these analyses, it was possible to observe that lupeol forms hydrogen bonds with the amino acids HIS462 and ARG463. In addition, it forms alkyl and pi-alkyl interactions with the amino acids TYR118, LEU121, PHE126, ILE131, TYR132, PHE228, LEU376, ILE465 and MET502 (Figs 4 and S2). The target-fishing results showed that lupeol interacts with targets that affect lipid synthesis.

### Lupeol has a nematicidal activity against *C. elegans*

**Fig. 5.**
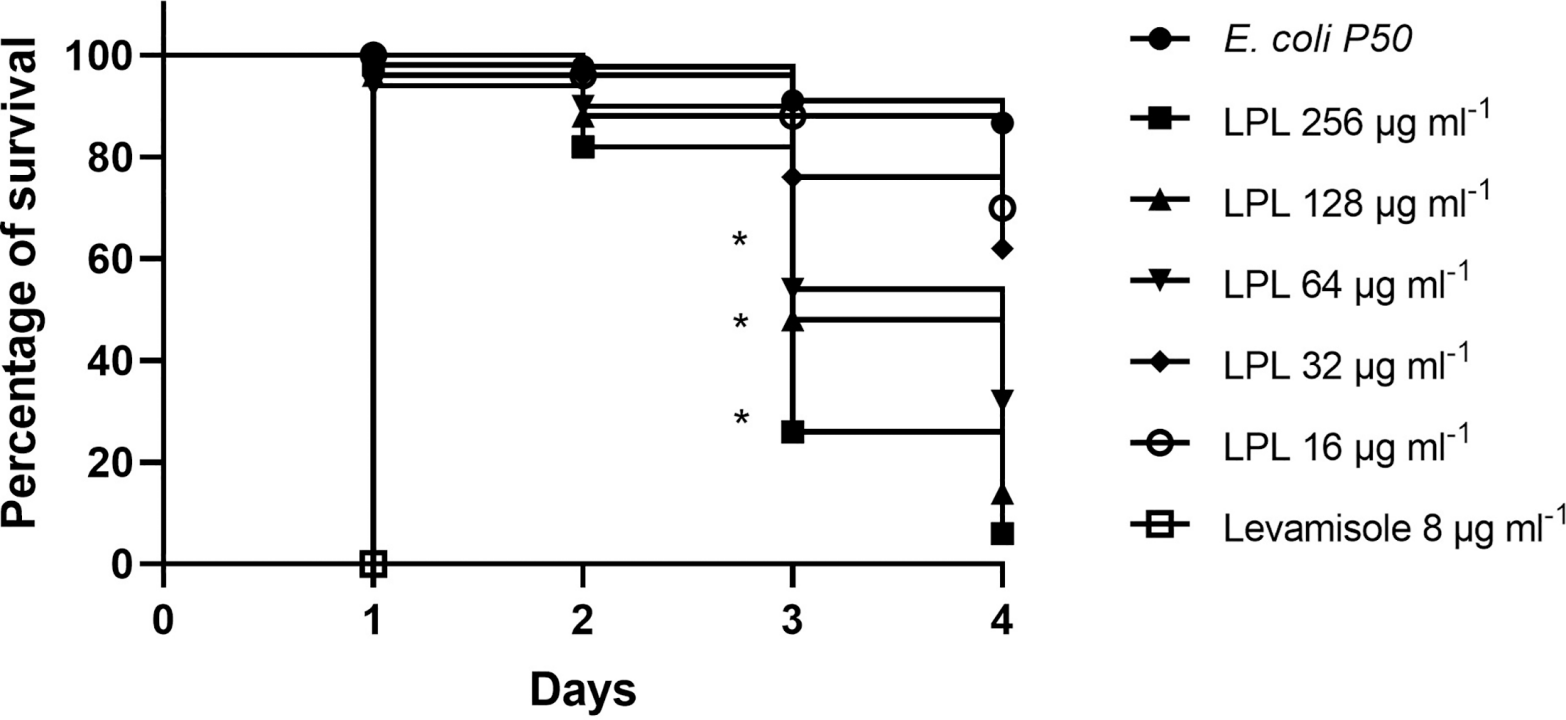
Survival curve of *Caenorhabditis elegans* nematodes in the L4 stage after exposure to the compound lupeol. Each group of nematodes was exposed to lupeol at different concentrations over 96 h. Dead nematodes were counted and removed every 24 h. The negative control consisted of nematodes that fed on *Escherichia coli* OP50, and the positive control consisted of nematodes exposed to levamisole. *Indicates statistically significant differences (*P*<0.05) compared with the negative control.

In the nematicidal activity assay of *C. elegans*, variable mortality was observed among the concentrations used, with higher percentages 72 h after exposure to lupeol. The analysis of the mortality rates indicated that throughout the entire observation period, significantly different survival times were found in the survival curves studied (*P*<0.0001). At the end of the 96 h evaluation, lupeol was capable of causing up to 86% mortality at the concentration of 128 µg ml^−1^ and reached a maximum mortality percentage of 94% at the maximum concentration (256 µg ml^−1^). The nematicidal effect of lupeol was statistically significant from the concentration of 64 µg ml^−1^ in comparison with the negative control (*P*<0.0025) (Figs 5 and S3).

## Discussion

Lupeol has been widely studied in recent years in many contexts, but a gap exists regarding its antifungal potential [18,21,38]. Our choice of lupeol comes from the observation of previous screening studies [41,43], in which they observed that the compound exhibits antifungal activity, including against *C. albicans*. Despite this, we found no studies evaluating its effect on the *C. parapsilosis* species complex. Therefore, in this study, we investigated the antifungal potential against the planktonic forms and mature biofilms of these yeasts.

As for its antimicrobial effect, different studies have shown that lupeol has activity against protozoa and bacteria, including micro-organisms with high pathogenic potential, such as methicillin-resistant *Staphylococcus aureus* [44,46]. As for its antifungal activity, lupeol has been shown capable of inhibiting up to 90% of *Penicillium notatum* growth at a concentration of 200 µg ml^−1^ [47]. Its inhibitory effect on fungal growth has also been demonstrated versus other species, such as *Sporothrix shenckii complex* and *Microsporum canis* [41,48]. Against *Candida* species, its activity on *C. albicans* was considered low [41,43]. Another study evaluating the effect of lupeol isolated from the leaves of *Curtisia dentata* obtained MIC of 940 µg ml^−1^ on *C. albicans*, with a fungicidal effect from 3750 µg ml^−1^ [42]. We observed lupeol MICs ranging from 64 to 512 µg ml^−1^ for the three species of the * C. parapsilosis* complex. In recent years, *C. parapsilosis* complex species have been the focus of several studies due to their increased prevalence in hospital infections in different regions of the world; moreover, the growing concern regarding antifungal resistance, especially to azole derivatives. These aspects justify expanding the investigation of antifungal compounds against these species [49,52]. In a nutshell, lupeol has shown effects against various micro-organisms, including the *C. parapsilosis* species complex, highlighting its potential as an antifungal compound.

It was observed in this research that lupeol reduces the biomass and metabolic activity of mature biofilms produced by the three species of the *C. parapsilosis* complex, which is more expressive even in lower concentrations. The reduction in metabolic activity was significantly more pronounced than the reduction in biomass after exposure to lupeol. This may indicate that debris from dead cells remained attached to the biofilm or the well. A previous study demonstrated the ability of this compound to reduce the biofilm biomass by up to 43% for the species *Macrophomina phaseolina*, a phytopathogenic filamentous fungus [53]. However, no studies were found regarding its effect on metabolic activity. It is known that the formation of biofilm is the main challenge in combating micro-organisms [54]. As presented by CLSM, lupeol decreased the total average area for both evaluated species, suggesting that it promotes the weakening of the biofilm of *C. parapsilosis* complex species. Therefore, in addition to its antifungal activity, lupeol, by promoting the weakening of biofilm, probably makes the cells more susceptible to the action of classical antifungal drugs. However, more experiments are needed to prove this hypothesis.

In general, research on the mechanism of action of lupeol against bacteria and fungi is scarce. Our results point to the potential fungistatic application of lupeol against *C. parapsilosis* complex species. This effect can be explained, at least in part, by its interaction with the enzyme sterol 14α-demethylase (CYP451), as observed in molecular docking analyses. This enzyme, which is part of cytochrome P450, is necessary for the biosynthesis of sterols in eukaryotic cells, being responsible for removing the 14α-methyl group from the first cyclized sterol precursor, lanosterol, which initiates the advancement of the pathway towards its final product, ergosterol in fungi [55]. This enzyme is one of the main targets of antifungal drugs, such as azoles, which block ergosterol biosynthesis precisely by inhibiting its activity [56,57]. With regard to the lupeol binding site on the enzyme, it binds to an important region of this enzyme, described by Monk *et al*. [58] as a ligand binding pocket, a region to which itraconazole also binds to carry out its mechanism of action. Thus, based on this preliminary analysis, we hypothesized that lupeol might modify the steps following lanosterol demethylation and that this event could culminate in both impairment of ergosterol biosynthesis and accumulation of sterols that are toxic to fungal cells, similar to what occurs with azoles [57,59]. The occurrence of resistance to azoles by structural enzymatic modifications could also lead to resistance to lupeol. However, lupeol can affect multiple biological targets as described in phytochemicals [32,60].

Nematodes, whether parasitic to plants, animals or humans, represent an important group of pathogens since they can cause significant losses to farmers and livestock breeders as well as threaten public health [61]. The search for products of natural origin that have activity against these agents is increasingly necessary due to concerns about environmental impacts and human health. Based on several studies, *C. elegans* has been established as an effective and economical model for the discovery of new broad-spectrum anthelmintics [61,63]. In this study, we evaluated the nematicidal activity of lupeol at different concentrations and observed that the compound exhibits a potent effect against *C. elegans* larvae. The maximum observation time was 96 h, a period in which lupeol (256 µg ml^−1^) caused 95% of the larval mortality. Corroborating our findings, in a study carried out by Shai *et al*. [64], *Curtisia dentata* (Cornaceae) leaf extracts and compounds isolated from leaves of this plant, such as betulinic acid and lupeol, inhibited motility of parasitic and free-living nematodes. In addition, other studies evaluating different classes of terpenes, such as carvacrol, geraniol, eugenol, thymol, neurolidol and limonene, also demonstrated their nematicidal activity [65,66]. We hypothesize that the nematicidal activity of lupeol may be associated with more than one specific target, similar to what occurs with other phytochemicals [67]. Furthermore, we suggest that the reduction in larval motility may indicate that the compound acts by affecting the nervous system of nematodes. Briefly, our research demonstrates the antiparasitic potential of lupeol and, therefore, merits further study of this terpene as a nematicide for the control of parasitic nematodes. Future research should involve investigating its nematicidal activity against parasitic species of relevance, such as *Haemonchus contortus*, as well as its effect on species resistant to conventional anthelmintics. The use of lupeol for controlling parasitosis is noteworthy due to its potential as an affordable effective alternative or enhancer of anthelmintic drugs.

In summary, this study demonstrated the antifungal potential of lupeol against *C. parapsilosis* species complex, both in planktonic form and mature biofilms. Data further show that this effect is possibly associated with its interaction with the enzyme 14α-demethylase and the ergosterol biosynthesis pathway. In addition, lupeol demonstrated a nematicidal potential.

## supplementary material

10.1099/jmm.0.001976 Uncited Fig. S1.

10.1099/jmm.0.001976 Uncited Fig. S2.

10.1099/jmm.0.001976 Uncited Fig. S3.

## Abbreviations

ATCC: American Type Culture Collection;
BHI: brain heart infusion agar
CLSI: Clinical and Laboratory Standards Institute
CLSM: confocal laser scanning microscopy
LPL: lupeol
MTT: 3-[4,5-dimethylthiazol-2-yl]-2,5 diphenyl tetrazolium bromide
NCBI: National Center for Biotechnology Information
NGM: nematode growth medium
