# Supplementary figures and images for: Antimicrobial and antiparasitic potential of lupeol: antifungal effect on the Candida parapsilosis species complex and nematicidal activity against Caenorhabditis elegans
Source: J Med Microbiol. 2025 Mar 7;74(3):001976. doi: 10.1099/jmm.0.001976 (PMC11936342; doi:10.1099/jmm.0.001976)

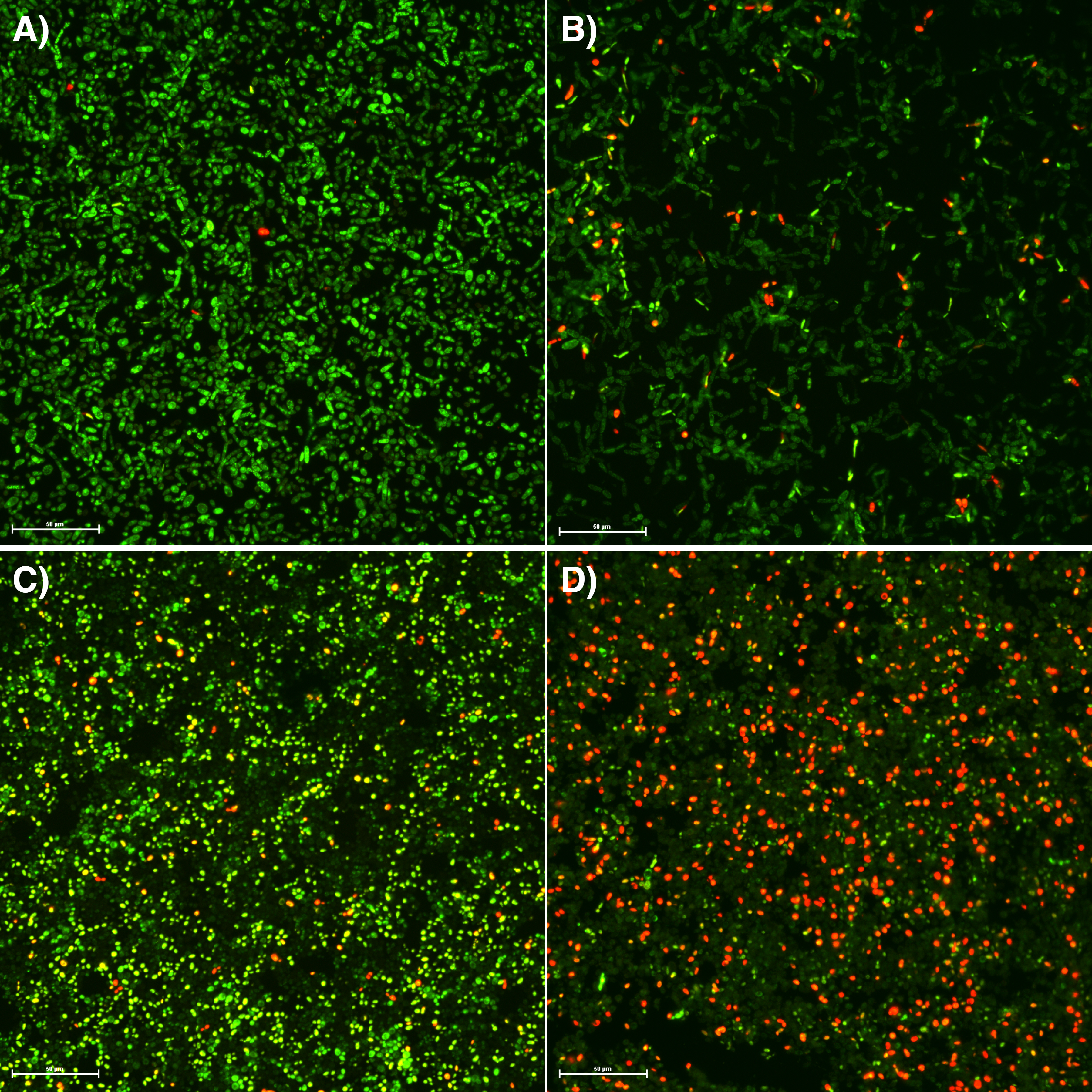

Supplement: Uncited Fig. S1. [file jmm-74-01976-s001.png]

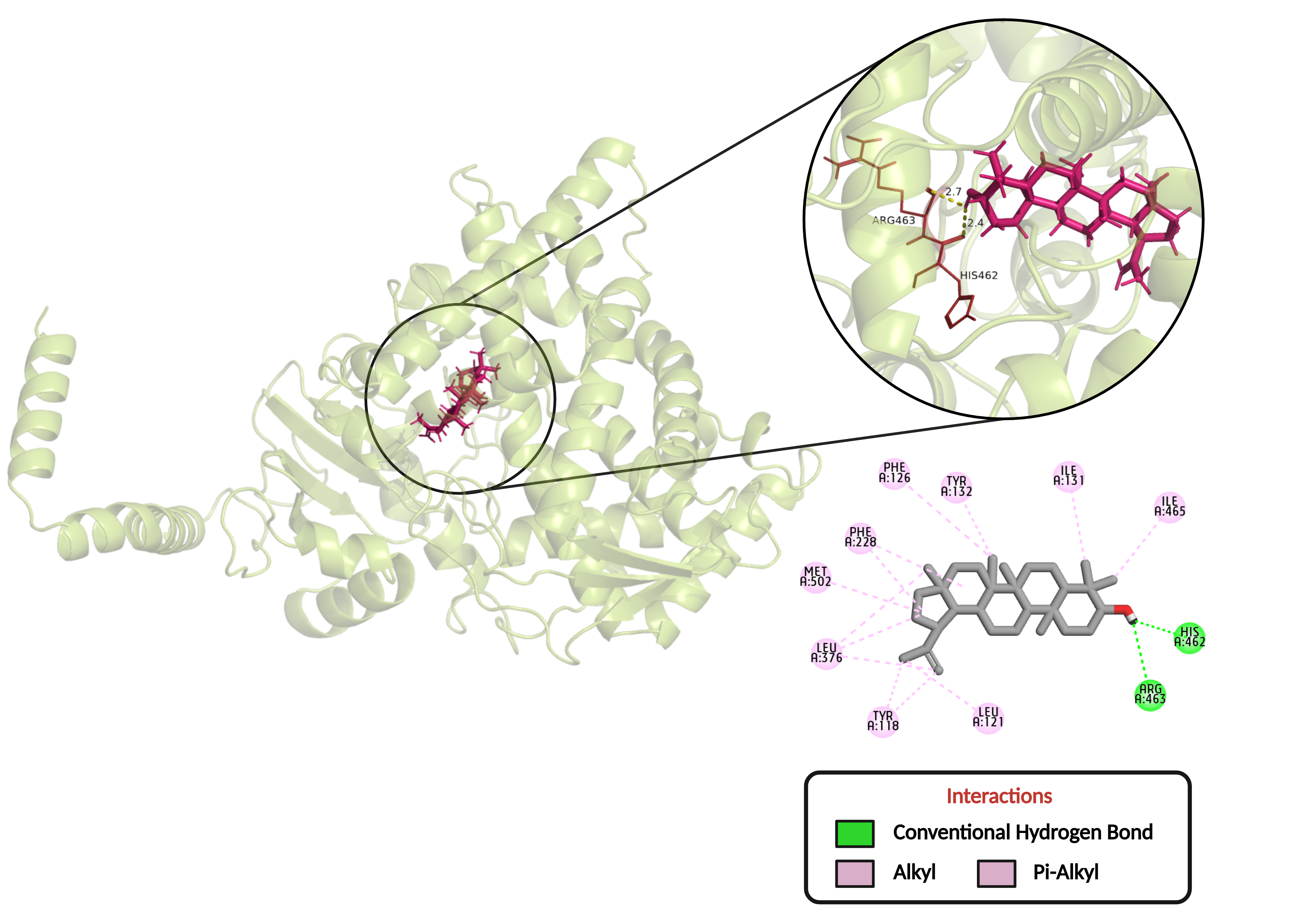

Supplement: Uncited Fig. S2. [file jmm-74-01976-s002.png]

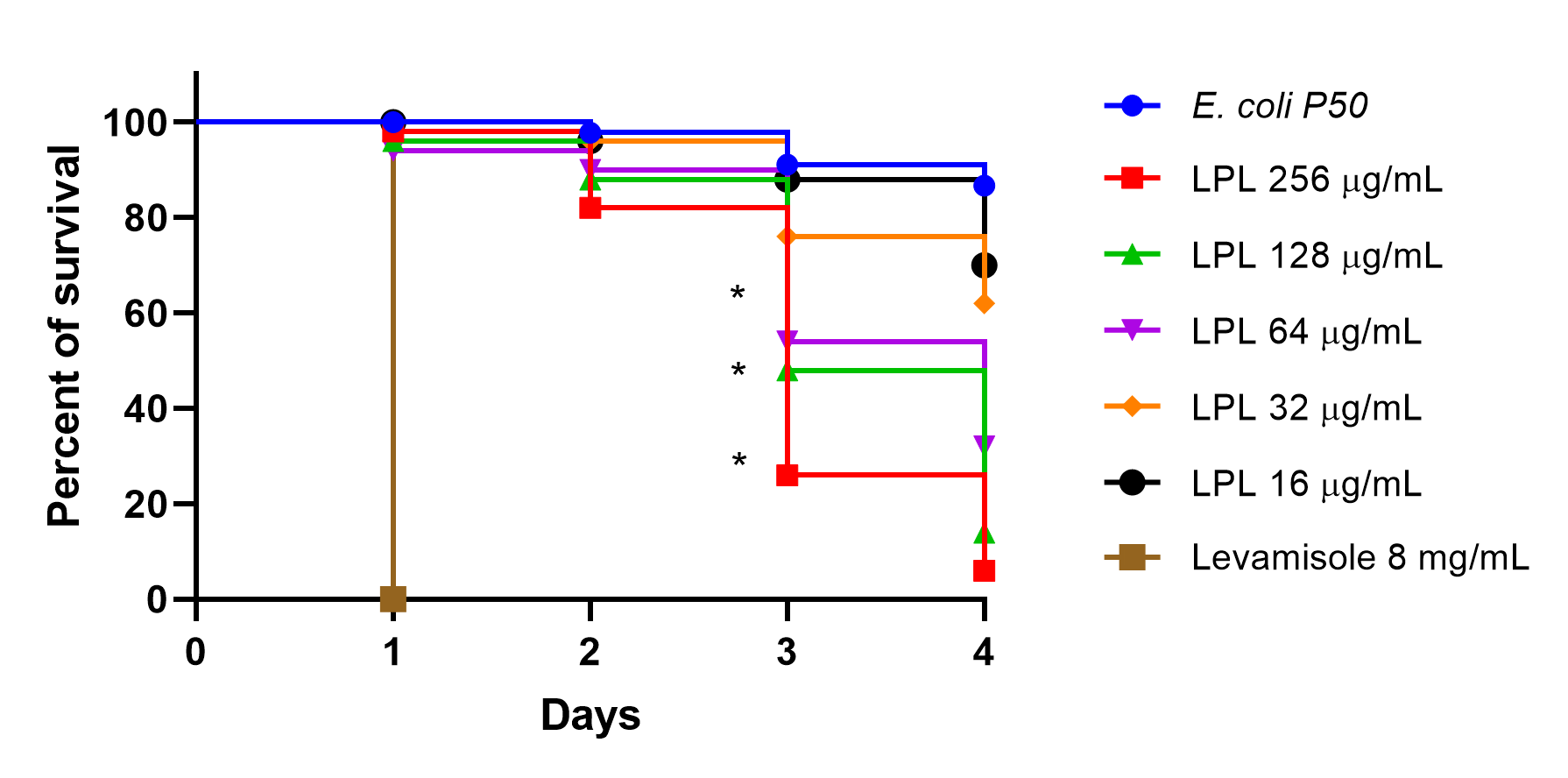

Supplement: Uncited Fig. S3. [file jmm-74-01976-s003.tif]
